# Obesity Drives an Oral Microbiota Signature of Female Patients with Periodontitis: A Pilot Study

**DOI:** 10.3390/diagnostics11050745

**Published:** 2021-04-21

**Authors:** Charlotte Thomas, Matthieu Minty, Thibault Canceill, Pascale Loubières, Vincent Azalbert, François Tercé, Camille Champion, Rémy Burcelin, Pierre Barthet, Sara Laurencin-Dalicieux, Vincent Blasco-Baque

**Affiliations:** 1 INSERM UMR 1297 Inserm, Institut des Maladies Métaboliques et Cardiovasculaires (I2MC), Avenue Jean Poulhès 1, CEDEX 4, 31432 Toulouse, France; charlotte.thomas@inserm.fr (C.T.); matthieu.minty@inserm.fr (M.M.); pascale.loubieres@inserm.fr (P.L.); vincent.azalbert@inserm.fr (V.A.); francois.terce@inserm.fr (F.T.); camille.champion@inserm.fr (C.C.); remy.burcelin@inserm.fr (R.B.); 2 Faculté de Chirurgie Dentaire, Université Paul Sabatier III (UPS), 118 Route de Narbonne, CEDEX 9, 31062 Toulouse, France; thibault.canceill@sfr.fr (T.C.); barthet_pierre@orange.fr (P.B.); laurencin.s@chu-toulouse.fr (S.L.-D.); 3 Service d’Odontologie Rangueil, CHU de Toulouse, 3 Chemin des Maraîchers, CEDEX 9, 31062 Toulouse, France; 4 UMR CNRS 5085, CIRIMAT, Université Paul Sabatier, 35 Chemin des Maraichers, CEDEX 9, 31062 Toulouse, France; 5 Institut de Mathématiques de Toulouse, Université Paul Sabatier, 118 Route de Narbonne, CEDEX 9, 31062 Toulouse, France; 6 INSERM U1295, CERPOP, Epidémiologie et Analyse en Santé Publique, Risques, Maladies Chroniques et Handicaps, 37 Allées Jules Guesdes, 31000 Toulouse, France

**Keywords:** oral microbiota, obesity, periodontitis, dysbiosis, sex/gender

## Abstract

The aim of this study was to analyze the link between oral microbiota and obesity in humans. We conducted a pilot study including 19 subjects with periodontitis divided into two groups: normo-weighted subjects (NWS) with a body mass index (BMI) between 20 and 25 (*n* = 9) and obese subjects (OS) with a BMI > 30 (*n* = 10). Obesity was associated with a poor oral health status characterized by an increased number of missing teeth and a higher score of periodontal-support loss associated with dysbiotic oral microbiota (39.45 ± 3.74 vs. 26.41 ± 11.21, *p* = 0.03 for the Chao 1 index). Oral microbiota taxonomic analysis showed that the abundance of the *Capnocytophaga* genus was higher (2.47% ± 3.02 vs. 0.27% ± 0.29, *p* = 0.04) in OS compared to NWS. Obese females (OF) were characterized by an increase in the *Streptococcus* genus (34.12% ± 14.29 vs. 10.55% ± 10.42, *p* = 0.05) compared to obese males (OM), where the *Neisseria* genus was increased (5.75% ± 5.03 vs. 58.05% ± 30.64, *p* = 0.008). These first data suggest that sex/gender is determinant in the link between oral dysbiotic microbiota and obesity in patients with periodontitis. Our results could lead to recommendations concerning therapeutic strategies for obese patients with periodontitis following the sex/gender.

## 1. Introduction

Obesity is recognized as a major public health problem. According to the World Health Organization, in 2017, 1.9 billion people were considered overweight, of which 650 million were obese which represents 39% of the adult population. In France, the results of the latest national ObÉpi survey conducted in 2012 showed a continuous increase in overweight and obesity among adults over 18 years of age with 15% of obese people, i.e., almost twice as many as in 1997 (8.5%) [1]. This increase is also found in the worldwide population in industrialized countries as well as in regions such as Sub-Saharan Africa that were considered spared until now. Nowadays, the evolution of this pathology is considered as pandemic with a clear inflammatory character which in turn increases the risk of development of other cardio-metabolic diseases such as hypertension, atherosclerosis, and type 2 diabetes. The causes of obesity are multifactorial and include lifestyle, diet, environment, and genetic factors. However, these etiologies are insufficient to explain its worldwide expansion and new research is required to identify other parameters. Recent data showed that a dysbiotic intestinal microbiota is involved in the development of obesity [2,3] and characterizes obese people [4]. Dysbiosis is defined by a qualitative and quantitative imbalance of the flora that can result in a decrease in bacterial diversity and/or an excess of certain pathogens. Obese people present an increase in the percentage of the Firmicutes phylum (Gram-positive) and a decrease in the Bacteroides phylum (Gram-negative) [5]. Moreover, obese people are also characterized by a dysbiotic oral microbiota with an increased Firmicutes-to-Bacteroidetes ratio [6]. The salivary microbiota contains 10^10^ bacteria and between 300 and 400 different species. Oral dysbiosis leads to an organizational change of this ecosystem that can potentiate the effect of particular species like anaerobic Gram-negative bacteria [7,8,9]. Interestingly, an increase in these Gram-negative bacteria is also associated with the development of a specific oral pathology: periodontitis. Periodontitis is a non-communicable, chronic inflammatory disease of infectious origin that causes irreversible destruction of the tooth surrounding tissues, ultimately leading to tooth loss [10,11]. The positive association between obesity and periodontal disease has been demonstrated in numerous clinical and epidemiological studies. Obese people were often associated with a lower socio-economic status compared to normo-weighted people. Low socioeconomic status is also associated with poor oral health and an increased risk of developing periodontal diseases [12,13,14]. Recently, the new classification of Periodontal and Peri-implant Diseases and Conditions (2017 European Federation of Periodontology and American Academy of Periodontology Consensus Report) highlights the predominant role of obesity in periodontal attachment loss via periodontal inflammation [15,16]. Obese people have a higher risk of developing severe periodontitis and, reciprocally, a higher body mass index (BMI) is observed in subjects with periodontitis [17,18,19]. According to the current literature, several mechanisms support the “bi-directional” relationship between obesity and periodontitis [20]. Some authors have implied that periodontitis and its dysbiotic microbiota may have a link with systemic diseases such as obesity [21,22,23]. In obese rats, adipose tissue inflammation was significantly aggravated by periodontitis with an increased expression of pro-inflammatory cytokines such as Interleukin-6 (IL-6) and Tumor Necrosis Factor α (TNF-α) [24,25]. Gram-negative oral bacteria and meta-factors, like lipopolysaccharide (LPS), in fatty tissues can induce a chronic, low-grade inflammatory response by macrophages and an increased production of pro-inflammatory adipokines in adipocytes [26]. This inflammatory state causes the proliferation of adipocyte precursors, predisposing to obesity [27].

As in the intestine, salivary dysbiosis can lead to the passage of Gram-negative bacteria or virulence factors through the oral mucosa, resulting in low-grade local and systemic inflammation called metabolic endotoxemia. Bacterial translocation from the mouth occurs via an increase in epithelial intercellular permeability induced by periodontitis. This endotoxemia associated with a chronic immuno-inflammatory reaction may contribute to the development and/or aggravation of metabolic disorders. However, research on the identification of causal oral bacteria species is still scarce. Different proportions of certain bacterial species have been observed, but their causality and mechanisms of action in obesity remain to be clarified. Thus, the link between obesity and dysbiotic oral microbiota needs to be further explored to identify a potential oral microbiota signature in obesity. The principal aim of this study was to analyze the oral microbiota status of obese subjects in relation to periodontitis. For this matter, we set up a pilot cohort study of 19 patients with periodontitis divided into two groups: normo-weighted subjects with a body mass index (BMI) between 20 and 25 (*n* = 9) and obese subjects with a BMI > 30 (*n* = 10), with an equivalent male-female ratio. Our results show that sex/gender plays a role in the oral microbiota signature of obesity in subjects with periodontitis.

## 2. Materials and Methods

We followed the STROBE statement guidelines for reporting observational studies [28]. The observational study was approved by the Commission Éthique du Département de Médecine Générale de Midi Pyrénées (local medical ethics committee).

### 2.1. Study Design

We conducted a cross-sectional study between January and February 2020 in Toulouse’s Public Teaching Hospital (CHU de Toulouse, France) including patients who were consulting for periodontal reasons. All of the participants gave their informed consent for participation in this study and agreement for an oral examination and saliva sampling for biological analyses.

### 2.2. Settings

The study was conducted in the Toulouse’s Public Teaching Hospital Periodontology consult (CHU de Toulouse, France) by two trained and calibrated investigators (C.T., S.L.-D.). The inclusion was proposed to every patient meeting inclusion criteria between January and February 2020 with no follow-up planed in the field of the study. As this study is non-interventional, the patient’s periodontal treatment was not different whether they consented or not to participate.

### 2.3. Participants

We included in this study adult patients with periodontitis who were able to understand and sign the consent form. In order to explore the differences between normo-weighted people and obese people, we excluded under-weighted patients with a BMI lower than 20 (kg/m^2^) and those over-weighted with a BMI between 25 (kg/m^2^) and 30 (kg/m^2^). We excluded the patients who presented diabetes (in order to purely explore obesity), liver diseases or steatosis (with an etiology of viral infections and autoimmune disease), chronic viral infections (Human immunodeficiency virus (HIV), Hepatitis B virus (HBV), Hepatitis C virus (HCV), and mononucleosis), chronic renal failure (creatinine clearance less than 60 mL/min), chronic or acute gastrointestinal diseases, history of gastrointestinal surgery modifying the anatomy, and those who were under anti-diabetic treatment or Proton Pump Inhibitors (PPI) treatment or had taken prebiotics, probiotics, or antibiotics in the month before the inclusion. Pregnant or breastfeeding women and people under guardianship or curatorship were also excluded. The participants were divided into two groups according to their BMI between 20 and 25 or over 30.

### 2.4. Variables and Data Measurements

The main outcome of this study was to analyze the oral microbiota’s taxonomic composition in both obese and normal weighted patients’ groups. Sampling was performed during the inclusion consultation which was conducted as following:

#### 2.4.1. Medical and Socio-Demographic Characteristics

Medical and dental history were assessed (associated pathologies, drugs, level of stress scored on a 10-points scale) as well as lifestyle and nutritional behavior of the patients, which were recorded from a questionnaire. This enabled us to focus on oral hygiene habits: frequency of visits to the dental surgeon and frequency and type of materials used (i.e., manual or electric toothbrush, mouthwash, interdental brushes).

#### 2.4.2. Oral Health Characteristics

For each patient, complete oral and periodontal examinations were carried out. A PCP15 probe was used for full-mouth periodontal charting (6 measurements per tooth) to determine probing depth, periodontal attachment loss, bleeding on probing (BOP) index, plaque index (PI), and score of periodontal-support loss in relation to the patient’s age and periodontal diagnostic (Stage and Grade). The decay-missing-filled (DMF) index and radiological exam were also conducted. Two qualified periodontists (C.T., S.L.-D.) conducted the oral health examinations. They were calibrated for full-mouth periodontal charting and periodontal assessment (probing depth, periodontal attachment loss, and BOP) before the launch of the study with an overall inter-rater kappa reaching 0.9. Prior to testing, the examiners verified that they had exactly the same protocol for the clinical examination focusing on both caries and periodontal status.

#### 2.4.3. Oral Microbiota Analysis

Unstimulated saliva was extracted and collected into sterile tubes on the day of the oral examination. Samples were then frozen into liquid nitrogen and stored at −80 °C for taxonomic analysis of the oral microbiota. Total DNA was extracted from frozen saliva using a Qiamp Cador Pathogen Mini kit (QIAGEN ref 54106, Les Ulis, France) according to manufacturer’s recommendations. Then, hypervariable V2 to V4 regions of the 16S bacterial ribosomal DNA (16S rDNA) were analyzed as previously described [29,30]. The Miseq sequencing of the samples was performed by Vaiomer (Labège, France).

### 2.5. Data Management and Analysis

#### 2.5.1. Bioinformatics Pipeline

The 16S-targeted metagenomic MiSeq reads from oral samples were analyzed using the bioinformatics pipeline established by Vaiomer (Labège, France) based on the FROGS v1.4.0 tool [31]. Briefly, after demultiplexing of the bar-coded Illumina paired reads, R1 and R2 read sequences were trimmed off, respectively, 10 and 40 bases to remove lower quality bases. Clean reads were paired for each sample independently into longer fragments using FLASH. The resulting amplicons were further cleaned by removing unspecific amplifications, i.e., fragments shorter than 350 and longer than 500 bases or without the two PCR primer sequences (10% mismatches allowed). Operational taxonomic units (OTUs) were produced via single-linkage clustering using the Swarm algorithm v2.1.6 in two passes: the first pass was a clustering with an aggregation distance equal to 1 and the second pass with an aggregation distance equal to 3. The OTUs identified as chimera (with VSEARCH v1.9.5) in all samples in which they were present were removed. The OTUs with abundance lower than 0.005% of the whole dataset abundance were removed. Finally, taxonomic assignment was performed in order to determine community profiles using the Blast+ v2.2.30+ against the Silva 128 Parc databank.

#### 2.5.2. Statistical Analyses

**Figure 1:**
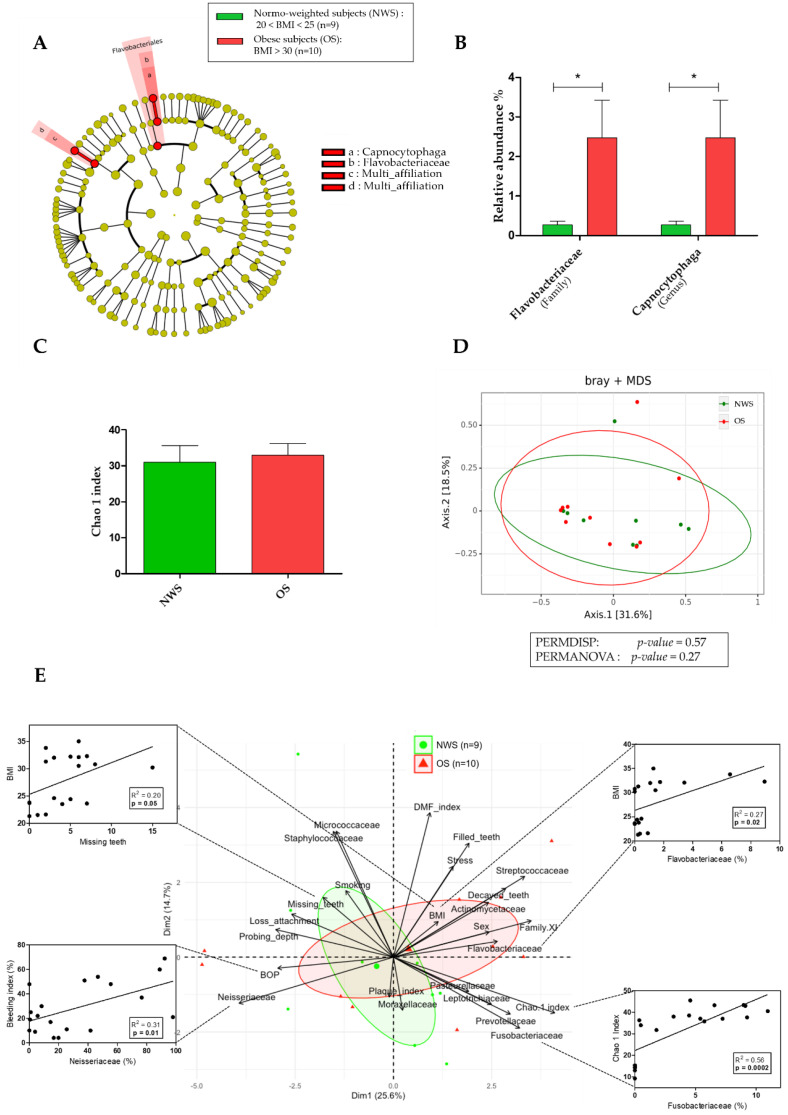
Oral microbiota in obese subjects (OS; *n* = 10) compared to normo-weighted subjects (NWS; *n* = 9). (**A**) Linear discriminant analysis effect size (LEfSe) analysis-based cladogram for oral microbiota; (**B**) Relative abundance (%) for taxonomic family and genus, identified with significant differences in saliva microbiota; (**C**) Chao 1 index representation of alpha diversity; (**D**) Bray–Curtis index representation of the beta diversity between; (**E**) Principal Component Analysis (PCA) between dominant bacterial genera from oral microbiota and oral clinical parameters and Pearson’s correlation analysis. Data as mean ± SD, * *p* < 0.05, unpaired Mann–Whitney test.

**Figure 2:**
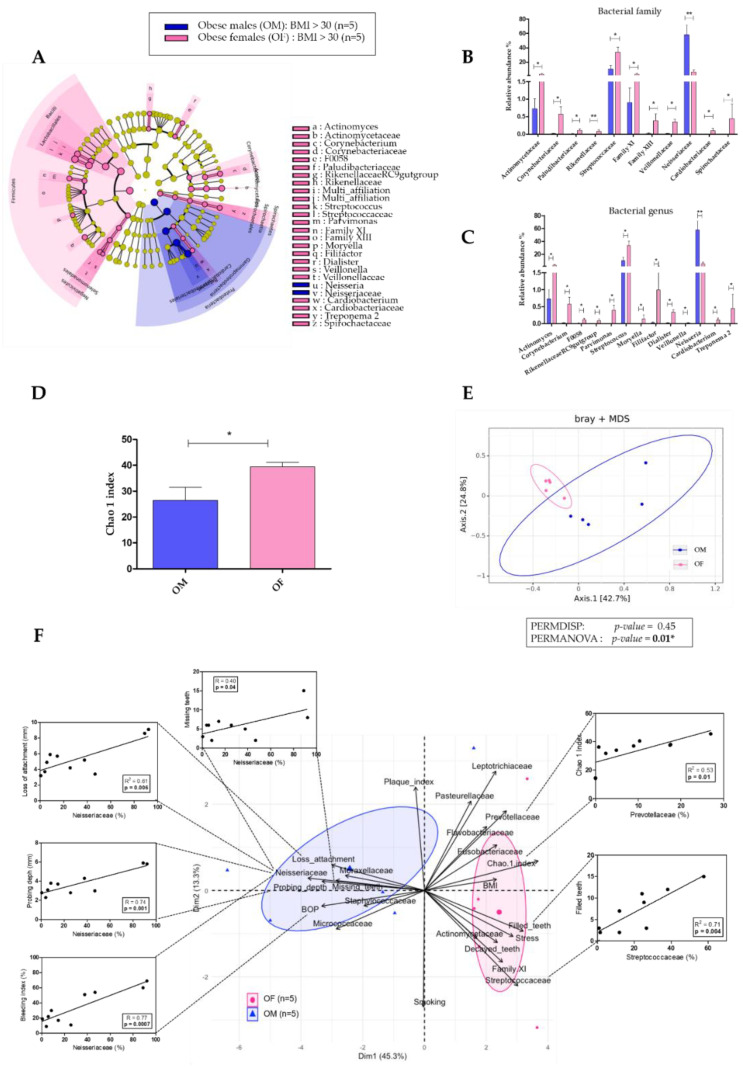
Comparison of oral microbiota between obese males (OM; *n* = 5) and obese females (OF; *n* = 5). (**A**) Linear discriminant analysis effect size (LEfSe) analysis-based cladogram for oral microbiota; (**B**,**C**) Relative abundance (%) for taxonomic family and genus, identified with significant differences in saliva microbiota; (**D**) Chao 1 index representation of alpha diversity; (**E**) Bray–Curtis index representation of the beta diversity between; (**F**) Principal Component Analysis (PCA) and Pearson’s correlation analysis between dominant bacterial genera from oral microbiota and oral clinical parameters. Data as mean ± SD, * *p* < 0.05, ** *p* < 0.01, unpaired Mann–Whitney test.

**Figure 3:**
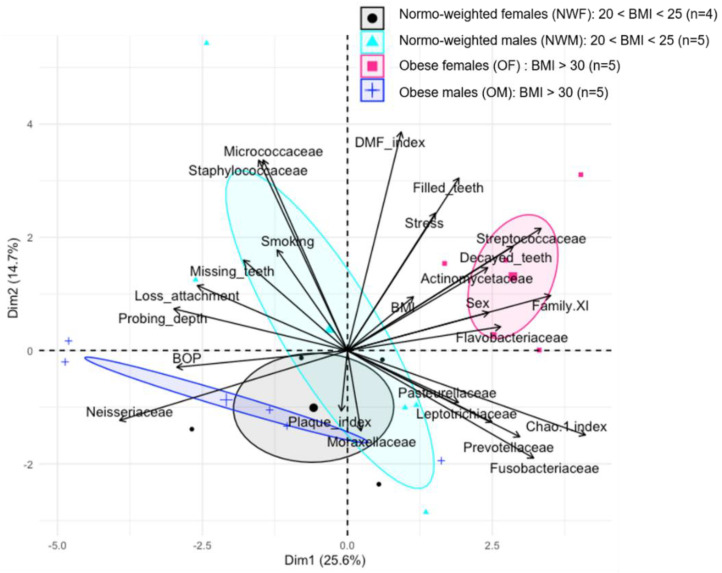
Principal Component analysis (PCA) between dominant bacterial families from oral microbiota and oral clinical parameters in 4 groups: normo-weighted females (NWF; *n* = 4), normo-weighted males (NWM; *n* = 5), obese females (OF; *n* = 5), and obese males (OM; *n* = 5).

**Figure 4:**
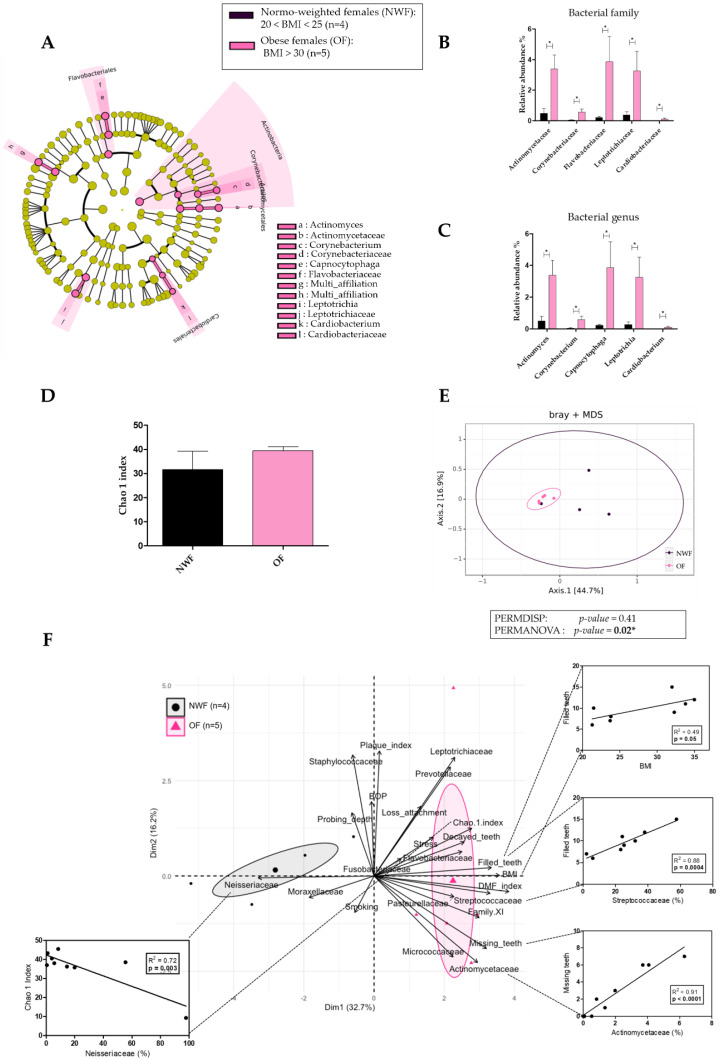
Comparison of oral microbiota between normo-weighted females (NWF; *n* = 4) and obese females (OF; *n* = 5). (**A**) Linear discriminant analysis effect size (LEfSe) analysis-based cladogram for oral microbiota; (**B**,**C**) Relative abundance (%) for taxonomic family and genus, identified with significant differences in saliva microbiota; (**D**) Chao 1 index representation of alpha diversity; (**E**) Bray–Curtis index representation of the beta diversity between; (**F**) Principal Component analysis (PCA) and Pearson’s correlation analysis between dominant bacterial genera from oral microbiota and oral clinical parameters. Data as mean ± SD, * *p* < 0.05, unpaired Mann–Whitney test.

Data were blinded to maintain participant confidentiality. The statistical analyses were corrected with the false discovery rate (FDR), a method of conceptualizing the rate of type I errors in null hypothesis testing when conducting multiple comparisons [32]. The bacterial diversity analyses (Chao1 alpha diversity and Bray–Curtis beta diversity) were performed using the Phyloseq v1.14.0 R package. The differential taxa analyses were conducted with the linear discriminant analysis effect size tool LEfSe [33] using default parameters (alpha parameter significance threshold set to 0.05 and the logarithmic LDA score cutoff set to 2.0). Unpaired Mann–Whitney tests were performed using GraphPad Prism (GraphPad Software, San Diego, CA, USA). A PERMANOVA test was performed with 999 permutations to assess the statistical significance of the difference between groups in beta diversity PCoA analyses (Figures 1D, 2E, and 4E). Next, we performed an exploratory data analysis by using principal component analysis (PCA) to identify which parameters characterized best the obese and normo-weighted subjects (Figure 1E, Figure 2F, Figure 3, and Figure 4F). Represented among those variables are bacterial groups identified by the cladogram in the oral microbiota and clinical parameters such as sex, BMI, stress, number of decayed, missing and filled teeth, smoking, plaque index (PI) and bleeding on probing (BOP), loss of attachment, and probing depth.

## 3. Results

### 3.1. Obese Subjects Present an Increased Periodontal Risk with More Dental Loss, Associated with a Greater Quantity of Capnocytophaga in Oral Microbiota Compared to Normo-Weighted Subjects

As reported in Table 1, the mean age of the subjects was not significantly different between obese and normo-weighted groups: 59.4 years old ± 11.65 with 50% of female (*n* = 5) in the obese group versus 57.11 years old ± 10.49 with 44% of females (*n* = 4) in the normo-weighted group. As the selection criterion, mean body weight (91.4 kg ± 9.38) and BMI (30.02 ± 1.48) of obese subjects (OS) were significantly higher than normo-weighted subjects (NWS) (67.33 kg ± 6.93 and 23.11 ± 1.29, respectively, *p* < 0.001 for both). No significant difference between OS and NWS was observed as far as stress score, nutritional behavior, and lifestyle habits were concerned.

To explore the link between obesity and oral health, different clinical parameters were analyzed (Table 1). The decay-missing-filled (DMF) index was similar between obese and normo-weighted subjects (14.11 ± 5.84 vs. 12.89 ± 6.23, *p* = 0.69). However, the number of missing teeth was significantly higher in obese patients (6.00 ± 3.77 vs. 2.44 ± 2.50, *p* = 0.03). Concerning the periodontal status, OS presented a significantly higher score of periodontal-support loss in relation to the patient’s age corresponding to periodontitis Grade C (1.03 ± 0.35 vs. 0.72 ± 0.23, *p* = 0.04) compared to NWS (Grade B). Periodontitis Grade C corresponds to the greatest speed of progression of the pathology. Finally, clinical attachment loss (5.39 mm ± 2.05 vs. 4.68 mm ± 1.29, *p* = 0.74), plaque index (16.8% ± 14.41 vs. 14.67% ± 14.56, *p* = 0.43), and bleeding on probing (34.2% ± 22.16 vs. 23% ± 17.73, *p* = 0.19) were not significantly different between OS and NWS.

To evaluate the association between oral microbiota and obesity, we performed a taxonomic analysis of the oral microbiota in both groups (Figure 1 and Supplementary Table S1). The relative abundance of the Flavobacteriaceae family (2.47% ± 3.02 vs. 0.27% ± 0.29, *p* = 0.04) and *Capnocytophaga* genus (2.47% ± 3.02 vs. 0.27% ± 0.29, *p* = 0.04) were higher in OS compared to NWS, and the *Capnocytophaga* genus was the only genus to be present in the Flavobacteriaceae family (Figure 1A,B). *Helicobacter* genus and *Helicobacter pylori* species were not detected in either group in oral microbiota. In addition, no difference was observed for the alpha diversity following the Chao 1 index (32.93 ± 10.45 vs. 30.94 ± 14.16, *p* = 0.93) and the beta diversity (*p* = 0.27) in the oral microbiota between obese and normo-weighted groups (Figure 1C,D).

To explore the interaction between clinical and microbial parameters related to obesity, we performed a multivariate analysis by Principal Component Analysis (PCA) (Figure 1E). We identified a specific cluster for each group of subjects. Pearson’s correlation analysis was then performed to estimate the relationships between all the parameters that directly influence the group distribution. The relative abundance of Neisseriaceae was positively and significantly correlated with BOP index. Fusobacteriaceae abundance was positively and significantly correlated with microbiota diversity according to the Chao 1 index. The number of missing teeth and the relative abundance of Flavobacteriaceae were positively and significantly correlated with BMI, and we observed that the closest clinical parameter associated with Flavobacteriaceae was the sex/gender. Thus, we hypothesized a link between the sex/gender and the oral microbiota, and we analyzed the implication of the sex/gender within the group of obese subjects.

### 3.2. Obese Females Have a Higher Number of Decayed and Filled Teeth Compared to Obese Males Associated with a Dysbiotic Oral Microbiota

Besides the weight and height that were higher in obese males, overall general parameters were similar in both obese females (OF) and males (OM), except for the stress score significantly higher in females (6.40 ± 1.52 vs. 2.8 ± 1.92, *p* = 0.01) (Table 2).

In order to investigate gender-related differences in obese subjects, the oral health status was analyzed. OF had a higher DMF index than OM (18.50 ± 3.11 vs. 10.6 ± 5.12, *p* = 0.08) due to an increase in the number of decayed (2.25 ± 2.21 vs. 0, *p* = 0.04) and filled teeth (11.75 ± 2.50 vs. 3.40 ± 2.07, *p* = 0.01). However, concerning the periodontal status, OM presented seemingly more severe cases of periodontitis measured by the clinical attachment loss (6.10 mm ± 2.60 vs. 4.68 mm ± 1.20, *p* = 0.46), the score of periodontal-support loss in relation to the patient’s age (1.16 ± 0.40 vs. 0.90 ± 0.28, *p* = 0.24), the number of missing teeth (7.2 ± 4.86 vs. 4.80 ± 2.16, *p* = 0.59), the plaque index (21% ± 13.73 vs. 12.6% ± 15.32, *p* = 0.34) and bleeding on probing (BOP) index (49% ± 22.33 vs. 19.4% ± 7.64, *p* = 0.07) compared to OF.

We then analyzed the oral microbiota in both OF and OM. The relative abundance of many oral bacterial families was significantly higher in OF compared to OM (Figure 2A,B): Actinomycetaceae (3.38% ± 2.10 vs. 0.73% ± 0.62, *p* = 0.05), Corynebacteriaceae (0.57% ± 0.47 vs. 0.02% ± 0.03, *p* = 0.01), Paludibacteriaceae (0.11% ± 0.11 vs. 0.007% ± 0.02, *p* = 0.01), Rikinellaceae (0.08% ± 0.11 vs. 0.001% ± 0.001, *p* = 0.009), Streptococcaceae (34.12% ± 14.29 vs. 10.55% ± 10.42, *p* = 0.05), Family XI (3.44% ± 2.24 vs. 0.91% ± 0.91, *p* = 0.03), Family XIII (0.39% ± 0.43 vs. 0.02% ± 0.03, *p* = 0.01), Veillonellaceae (0.36% ± 0.16 vs. 0.02% ± 0.02, *p* = 0.01), Cardiobacteriaceae (0.11% ± 0.14 vs. 0.01% ± 0.01, *p* = 0.01), and Spirochaetaceae (0.45% ± 0.13 vs. 0% ± 0, *p* = 0.02) (Supplementary Table S2). The Neisseriaceae family was the only one significantly lower in OF compared to OM (6.63% ± 5.27 vs. 58.20% ± 30.47, *p* = 0.008). Higher relative abundance in OF was similarly observed for oral bacterial genera (Figure 2A,C): *Actinomyces* (3.38% ± 2.10 vs. 0.73% ± 0.62, *p* = 0.05), *Corynebacterium* (0.57% ± 0.47 vs. 0.02% ± 0.03, *p* = 0.01), *F*0058 (0.11% ± 0.11 vs. 0.007% ± 0.01, *p* = 0.01), *Parvimonas* (0.40% ± 0.33 vs. 0.006% ± 0.005, *p* = 0.01), *Streptococcus* (34.12% ± 14.29 vs. 10.55% ± 10.42, *p* = 0.05), *Moryella* (0.14% ± 0.24 vs. 0% ± 0, *p* = 0.02), *Filifactor* (0.99% ± 1.18 vs. 0.03% ± 0.03, *p* = 0.01), *Dialister* (0.33% ± 0.15 vs. 0.02% ± 0.02, *p* = 0.01), *Veillonella* (0.02% ± 0.02 vs. 0.001% ± 0.002, *p* = 0.009), *Cardiobacterium* (0.11% ± 0.14 vs. 0.005% ± 0.007, *p* = 0.01), and *Treponema 2* (0.45% ± 0.13 vs. 0% ± 0, *p* = 0.02) (Supplementary Table S2). As for the Neisseriaceae family, the *Neisseria* genus was the only one significantly lower in OF compared to OM (5.75% ± 5.03 vs. 58.05% ± 30.64, *p* = 0.008). Moreover, the alpha diversity was significantly higher in OF compared to OM (39.45 ± 3.74 vs. 26.41 ± 11.21, *p* = 0.03 for the Chao 1 index) (Figure 2D), and the beta diversity was also significantly different (*p* = 0.01) (Figure 2E).

To identify clinical and microbiota parameters associated with sex/gender in obesity, we performed a Principal Component Analysis (PCA) and a Pearson’s correlation analysis (Figure 2F). The PCA reported that the relative abundance of Neisseriaceae (key bacterial family in OM) was positively and significantly correlated with parameters determining the periodontal status (number of missing teeth, probing depth, loss of attachment, and BOP index). The relative abundance of Streptococcaceae (a key bacterial family of OF) was positively and significantly correlated with the number of filled teeth.

More interestingly, when performing a Principal Component Analysis (PCA) separating the four groups (normo-weighted males, normo-weighted females, obese males, and obese females), we observed that obese females formed clearly an independent group (Figure 3).

### 3.3. Obesity Is Associated with an Impaired Oral Health and a Dysbiotic Oral Microbiota in Females

When analyzing overall general parameters in normo-weighted females versus obese females, we found no significant differences except for the selection criterion BMI (32.72 ± 1.73 vs. 22.58 ± 1.36, *p* = 0.01) and the mean body weight (86 kg ± 4.47 vs. 61 kg ± 2.45, *p* = 0.01) (Table 3). The DMF index (18.50 ± 3.11 vs. 8 ± 2.16, *p* = 0.02) as well as the number of missing (4.80 ± 2.16 vs. 0.25 ± 0.5, *p* = 0.01) and filled (11.75 ± 2.50 vs. 7.75 ± 1.70, *p* = 0.05) teeth of OF were significantly higher compared to normo-weighted females (NWF). Although no decayed teeth were observed in the normo-weighted group, the higher value in the obese group (2.25 ± 2.21) appeared non-significant (*p* = 0.06). In addition, no significant difference was observed for the other parameters relating to their periodontal status.

To explore the association between obesity and the oral microbiota in females, we compared microbiota differences between normo-weighted and obese females (Supplementary Table S3). The relative abundance of five oral bacterial families was significantly higher in OF compared to NWF: Actinomycetacea (3.38% ± 2.10 vs. 0.49% ± 0.62, *p* = 0.03), Corynebacteriaceae (0.57% ± 0.47 vs. 0.04% ± 0.06, *p* = 0.03), Flavobacteriaceae (3.86% ± 3.66 vs. 0.22% ± 0.15, *p* = 0.01), Leptotrichiaceae (3.25% ± 2.85 vs. 0.38% ± 0.42, *p* = 0.01), and Cardiobacteriaceae (0.11% ± 0.14 vs. 0.004% ± 0.003, *p* = 0.01) (Figure 4A,B). Similarly, the relative abundance of five oral bacterial genera was significantly higher in OF compared to NWF: *Actinomyces* (3.38% ± 2.10 vs. 0.49% ± 0.62, *p* = 0.03), *Corynebacterium* (0.57% ± 0.47 vs. 0.04% ± 0.06, *p* = 0.03), *Capnocytophaga* (3.86% ± 3.66 vs. 0.22% ± 0.15, *p* = 0.01), *Leptotrichia* (3.25% ± 2.85 vs. 0.26 ± 0.35, *p* = 0.01), and *Cardiobacterium* (0.11% ± 0.14 vs. 0.004% ± 0.003, *p* = 0.01) (Figure 4A,C). No difference was observed in the alpha diversity between both groups (31.69 ± 15.28 vs. 39.45 ± 3.74, *p* = 0.55 for the Chao 1 index) (Figure 4D). By contrast, the microbiota beta diversity was significantly different with a close clustering for obese females (*p* = 0.02) (Figure 4E).

To evaluate the link between obesity and all parameters (clinical and microbial) in females, we performed a Principal Component Analysis and a Pearson’s correlation (Figure 4F). PCA identified two distinct clusters represented by obese and normo-weighted females. We observed that Actinomycetaceae abundance was positively and significantly correlated with the number of missing teeth. Streptococcaceae abundance and BMI were positively and significantly correlated with the number of filled teeth. Furthermore, Neisseriaceae abundance was negatively and significantly correlated with microbiota diversity according to the Chao 1 index.

## 4. Discussion

In our study, we showed that obesity is associated with a poor oral health status characterized by the number of missing teeth, score of periodontal-support loss, and an increase in the *Capnocytophaga* genus (Flavobacteriaceae family). In addition, we established a relationship between sex/gender and dysbiotic oral microbiota in obese patients with periodontitis. Among them, obese males were characterized by an overall increase in the *Neisseria* genus associated with a more severe periodontal status compared to obese females, who presented an increase in the *Streptoccocus* genus usually more associated with the development of caries. Smoking is a well-known risk factor for developing periodontitis and oral dysbiosis. However, the significant clinical and microbiological differences presented in our pilot study are seemingly independent of the subject’s smoking status, since the proportion of smokers and non-smokers was equivalent in all groups.

Our data confirm evidence found in previous studies indicating that obese subjects present more severe cases of periodontitis than normo-weighted subjects, independently of their oral hygiene habits [17]. A recent study showed that obesity is also associated with a higher risk of tooth loss over five years [34]. In our study, obese subjects had significantly more missing teeth than normo-weighted subjects. Moreover, obese subjects have a greater speed of progression of their periodontitis (Grade C) than normo-weighted subjects (Grade B) with comparable stages [11]. Periodontal grading estimates the risk of developing periodontitis, the progression rate, as well as the frequency of periodontal recalls. Mechanisms supporting the “bi-directional” relationship between obesity and periodontitis have yet to be explored [12]. Obesity, characterized by a chronic low-grade inflammatory state, releases pro-inflammatory mediators from adipose tissues to blood, targeting periodontal tissues. In obese subjects with periodontitis, pro-inflammatory cytokines (Interleukin-1β (Il-1β), Interleukin-6 (Il-6), Tumor Necrosis Factor α (TNF-α)) were found in greater quantities in the blood and also in the crevicular gingival fluid compared to normo-weighted subjects with periodontitis [35,36]. In the crevicular gingival fluid, the level of pro-inflammatory adipokines (leptin, visfatin, and resistin) is also increased in obese subjects [37,38]. The increase of pro-inflammatory cytokines and adipokines in periodontal tissues promotes bone resorption processes caused by the recruitment of immuno-inflammatory cells [39,40]. The alteration of periodontal homeostasis is one of the first steps for shifting towards a dysbiotic oral microbiota, with an increase of Gram-negative bacteria implicated in periodontitis. Gram-negative bacteria disseminate and invade oral tissues through local inflammation.

In our study, we showed that obesity is associated with an overall significant increase of the *Capnocytophaga* genus in obese subjects compared to normo-weighted subjects, especially in obese females. *Capnocytophaga* is a Gram-negative bacteria implicated in periodontitis and it was also found in higher proportion in obese subjects [41]. Others like Wu et al. observed a higher proportion of certain families such as Prevotellaceae and Peptostreptococcaceae in the salivary microbiota of obese people [6]. *Tannerella forsythia* was also found in a higher proportion in obese subjects suffering from periodontitis [7]. However, few studies have explored the oral microbiota in obesity. Interestingly, in our study, both the Prevotellaceace and Tannerellaceae families were found to be more abundant, particularly in obese females. These oral bacteria activate innate immune defenses via Pathogen-Associated Molecular Patterns (PAMP) recognized by Pattern Recognition Receptors (PRR) of innate immune cells. PAMPs are represented by different surface molecules such as lipoteichoic acid (Gram-positive specific) or LPS (Gram-negative specific). PRRs include a group of receptors called Toll-Like Receptor (TLR) [42]. TLR-4 (LPS ligand) receptors, present in oral tissues, are involved in the response to oral Gram-negative bacteria. The interaction between TLRs and PAMPs leads to the activation of the MyD88 (Myeloid Differentiation protein 88) signaling pathway and then that of the transcription factor NF-kappa B (NF-kB). This chronic and local inflammatory state could promote systemic and local adipose inflammation through the translocation of live bacteria and bacterial metafactors such as LPS. Bacteria trigger the TLR-4 receptors located on the surface of the adipocytes [25,26,43,44]. In response to bacterial aggression, the secretory profile of adipokine is modified with a decrease in adiponectin (anti-inflammatory adipokine) and an increase in leptin, resistin, and Il-6 (pro-inflammatory adipokines) in adipocytes, increasing the inflammatory status of fatty tissues [26,45,46]. Bacterial translocation in adipose tissues also stimulates the proliferation of pre-adipocytes and macrophages [27].

We also found that obese females with periodontitis were characterized by a specific oral microbiota signature compared to obese males. Our results show that obese males present more severe cases of periodontal disease than obese females. Recent studies on subjects exempt from oral diseases have suggested the involvement of sex/gender in the relationship between obesity and oral microbiota [47]. Other studies confirm this higher prevalence and severity of periodontitis in males compared to females [48,49]. This more advanced periodontal disease is correlated with a significant increase in the abundance of the *Neisseria* genus in obese men. *Neisseria* is a Gram-negative oral bacteria that has been found in higher proportions in obese people [9]. A recent study by De Andrade et al. observed that the *Neisseria* genus was also impacted by sex/gender in obese males [47]. To our knowledge, only one study focused on the composition of oral microbiota of obese females suffering from periodontitis. Their results confirm ours in that they also found an increase in *Capnocytophaga* and *Streptococcus* genera [41]. In our study, obese females also tend to have a more severe carious status which could be correlated with the significant increase in the *Streptococcus* genus [50].

The originality of our study resides in the use of high throughput open-ended sequencing for oral microbiota analysis of patients suffering from periodontitis [51]. Indeed, many studies concerning subjects with periodontitis carry out a targeted microbiological analysis using genomic DNA probes [52]. A limited group of bacteria is then studied. High throughput sequencing overcomes the limitations encountered with DNA probes. It allows characterization, without a priori, of the whole oral microbiota of normo-weighted and obese subjects suffering from periodontitis. New bacterial associations, even with minority species, can be observed, allowing a better understanding of the different bacterial niches and their interactions within the oral microbiota. In our study, we have highlighted an increase of *Capnocytophaga* and *Neisseria* genera which are not considered as major periodontal pathogens. Nevertheless, they are Gram-negative bacteria, capable of translocating in the bloodstream. These opportunistic pathogens can be associated with periodontitis but also with a certain number of infectious diseases [53,54]. The metagenomic sequencing also enabled us to highlight very marked differences in microbiological profiles, particularly between obese and normal-weighted women, despite the low number of subjects. In fact, it will be necessary to confirm and refine the observations of this pilot study with a larger sample.

This study confirms the growing interest to determine the role played by the oral microbiota in obesity and the differences between males and females. Obese patients are at high risk of developing periodontal diseases. It is therefore essential to inform and educate these patients. Establishing healthier environmental measures (physical activity, balanced nutritional intake) associated with more frequent oral check-ups by dental surgeons are to be promoted in order to help limit the development of periodontitis. Moreover, within obesity, the clinical consequences being different between men and women with a greater severity of periodontal disease in males, increased comprehension of this periodontitis-obesity association will help to develop new targeted therapeutic strategies for the prevention and treatment of obese patients with periodontitis. Considering the patient’s profile, including gender, will allow for more personalized individual care.

## 5. Conclusions

In summary, we conducted a pilot study showing that sex/gender plays a major role in oral dysbiotic microbiota in obese subjects. The *Capnocytophaga* genus seems to emerge in this specific oral microbiota signature. Also, obese females with periodontitis are characterized by an increase of the *Streptococcus* genus compared to males. These results confirm the importance of sex/gender driven by obesity in dysbiotic oral microbiota in females.

## Figures and Tables

**Table 1:** General and oral parameters between normo-weighted subjects (NWS; *n* = 9) and obese subjects (OS; *n* = 10). Data as mean ± SD, * *p* < 0.05, *** *p* < 0.0001 unpaired Mann–Whitney test and Fisher test exact. Significant *p*-values have been highlighted in bold.

Parameters	Normo-Weighted Subjects (NWS) 20 < BMI < 25	Obese Subjects (OS) BMI > 30	*p*-Value
*n*	9	10	
Sex (number of women, %)	4 (44.44%)	5 (50%)	>0.99
Age (years)	57.11 ± 10.49	59.40 ± 11.65	0.57
Weight (Kg)	67.33 ± 6.93	91.40 ± 9.38	<0.001 ***
Height (cm)	170.56 ± 8.52	168.90 ± 10.17	0.51
BMI (kg/m^2^)	23.11 ± 1.29	30.02 ± 1.48	<0.001 ***
Smoking (number of smokers, %)	6 (67%)	6 (60%)	0.81
Stress on a scale from 0 to 10 (EVA)	3.78 ± 3.93	4.60 ± 2.50	0.46
Frequency of physical activity	1.39 ± 1.76	2.20 ± 2.97	0.76
Meal time (min)	31.89 ± 8.19	27 ± 4.83	0.09
DMF index	12.89 ± 6.23	14.11 ± 5.84	0.69
Number of decayed teeth	0 ± 0	1.00 ± 1.80	0.07
Number of missing teeth	2.44 ± 2.50	6.00 ± 3.77	0.03 *
Number of filled teeth	10.44 ± 4.15	7.11 ± 4.88	0.19
Score of periodontal-support loss in relation to the patient’s age	0.72 ± 0.23 (Grade B)	1.03 ± 0.35 (Grade C)	0.04 *
Probing depth (mm)	3.72 ± 1.02	3.75 ± 1.25	0.97
Loss of attachment (mm)	4.68 ± 1.29	5.39 ± 2.05	0.74
Plaque index (%)	14.67 ± 14.56	16.80 ± 14.41	0.71
Bleeding on probing (%)	23.00 ± 17.73	34.20 ± 22.16	0.19
Brushing frequency			
Once a day	1(1.11%)	3 (30%)	0.55
2 times a day	7 (77.78%)	5 (50%)	
3 times a day	1 (11.11%)	2 (20%)	
Dental check-up frequency			
Less than once every 2 years	0	1 (10%)	0.72
Once every 2 years	1 (11.11%)	1 (10%)	
Once a year	6 (66.67%)	4 (40%)	
2 times a year	2 (22.22%)	4 (40%)	

**Table 2:** Comparison of general and oral parameters between obese males (OM; *n* = 5) and obese females (OF; *n* = 5). Data as mean ± SD, * *p* < 0.05, unpaired Mann–Whitney test and Fisher test exact. Significant *p*-values have been highlighted in bold.

Parameters	Obese Males (OM) BMI > 30	Obese Females (OF) BMI > 30	*p*-Value
*n*	5	5	
Age (years)	56.80 ± 10.42	62.00 ± 13.41	0.59
Weight (Kg)	96.80 ± 10.26	86.00 ± 4.47	0.04 *
Height (cm)	175.60 ± 9.53	162.20 ± 5.45	0.01 *
BMI (kg/m^2^)	31.32 ± 0.85	32.72 ± 1.73	0.17
Smoking (number of smokers, %)	3 (60%)	3 (60%)	0.90
Stress on a scale from 0 to 10 (EVA)	2.80 ± 1.92	6.40 ± 1.52	0.01 *
Frequency of physical activity	2.40± 3.36	2.00 ± 2.91	0.82
Meal time (min)	26.00 ± 6.52	28.00 ± 2.74	0.81
DMF index	10.60 ± 5.12	18.50 ± 3.11	0.08
Number of decayed teeth	0 ± 0	2.25 ± 2.21	0.04 *
Number of missing teeth	7.20 ± 4.86	4.80 ± 2.16	0.59
Number of filled teeth	3.40 ± 2.07	11.75 ± 2.50	0.01 *
Score of periodontal-support loss in relation to the patient’s age	1.16 ± 0.40 (Grade C)	0.90 ± 0.28 (Grade B)	0.24
Probing depth (mm)	4.36 ± 1.48	3.14 ± 0.63	0.20
Loss of attachment (mm)	6.10 ± 2.60	4.68 ± 1.20	0.46
Plaque index (%)	21.00 ± 13.73	12.60 ± 15.32	0.34
Bleeding on probing (%)	49.00 ± 22.33	19.40 ± 7.64	0.07
Brushing frequency			
Once a day	3 (60%)	0	0.16
2 times a day	1 (20%)	4 (80%)	
3 times a day	1 (20%)	1 (20%)	
Dental check-up frequency			
Less than once every 2 years	1 (20%)	0	0.33
Once every 2 years	0	1 (20%)	
Once a year	1 (20%)	3 (60%)	
2 times a year	3 (60%)	1 (20%)	

**Table 3:** Comparison of general and oral parameters between normo-weighted females (NWF; *n* = 4) and obese females (OF; *n* = 5). Data as mean ± SD, * *p* < 0.05, unpaired Mann–Whitney test and Fisher test exact. Significant *p*-values have been highlighted in bold.

Parameters	Normo-Weighted Females (NWF) 20 > BMI > 25	Obese Females (OF) BMI > 30	*p*-Value
*n*	4	5	
Age (years)	56.00 ± 13.88	62.00 ± 13.41	0.31
Weight (Kg)	61.00 ± 2.45	86.00 ± 4.47	0.01 *
Height (cm)	164.50 ± 6.76	162.20 ± 5.45	0.62
BMI (kg/m^2^)	22.58 ± 1.36	32.72 ± 1.73	0.01 *
Smoking (number of smokers, %)	3 (75%)	3 (60%)	0.76
Stress on a scale from 0 to 10 (EVA)	5.25 ± 4.27	6.40 ± 1.52	0.62
Frequency of physical activity	1.00 ± 0.82	2.00 ± 2.91	0.89
Meal time (min)	30.00 ± 4.08	28.00 ± 2.74	0.41
DMF index	8.00 ± 2.16	18.50 ± 3.11	0.02 *
Number of decayed teeth	0 ± 0	2.25 ± 2.21	0.06
Number of missing teeth	0.25 ± 0.50	4.80 ± 2.16	0.01 *
Number of filled teeth	7.75 ± 1.70	11.75 ± 2.50	0.05 *
Score of periodontal-support loss in relation to the patient’s age	0.75 ± 0.23 (Grade B)	0.90 ± 0.28 (Grade B)	0.38
Probing depth (mm)	4.10 ± 1.24	3.14 ± 0.63	0.26
Loss of attachment (mm)	4.60 ± 1.69	4.68 ± 1.20	0.90
Plaque index (%)	12.00 ±11.22	12.60 ± 15.32	0.90
Bleeding on probing (%)	24.50 ± 18,12	19.40 ± 7.64	0.62
Brushing frequency			
Once a day	1 (25%)	0	0.68
2 times a day	2 (50%)	4 (80%)	
3 times a day	1 (25%)	1 (20%)	
Dental check-up frequency			
Less than once every 2 years	0	0	0.71
Once every 2 years	1 (25%)	1 (20%)	
Once a year	1 (25%)	3 (60%)	
2 times a year	2 (50%)	1 (20%)	

## Data Availability

The data presented in this study are available upon request from the corresponding author.

## Supplementary Materials

Supplementary materials can be found at https://www.mdpi.com/article/10.3390/diagnostics11050745/s1.
