# Supplementary material for: Obesity Drives an Oral Microbiota Signature of Female Patients with Periodontitis: A Pilot Study
Source: Diagnostics (Basel). 2021 Apr 21;11(5):745. doi: 10.3390/diagnostics11050745 (PMC8143370; doi:10.3390/diagnostics11050745)
Supplement: Supplementary file 1 [file diagnostics-11-00745-s001.zip › diagnostics-1160112-supplementary.pdf]

Table S1. Comparison between the Relative abundance (%) in oral microbiota of Normo-weighted subjects (NWS 20<BMI<25 (n=9) and Obese subjects (OS) BMI>30 (n=10). Data as mean ± SD, *p<0.05, **p< 0.001, ***p< 0.0001 unpaired Mann-Whitney test. Significant p-values have been highlighted in bold.

Relative abundance (%) in oral microbiota	Microbiota parameters	Normo-weighted subjects (NWS) 20<BMI<25 (n=9)	Obese subjects (OS) BMI>30 (n=10)	p-value
	α-diversity by the Shannon index	1.64 ±.0.80	1.81 ±.0.78	0.53
	α-diversity by the Chao 1 index	30.94 ± 14.16	32.93 ± 10.45	0.93
**Phylum**	Actinobacteria	9.77 ± 18.70	6.41 ± 2.83	0.15
	Bacteroidetes	15.42 ± 16.51	18.25 ± 12.66	0.83
	Epsilonbacteraeota	1.04 ± 1.21	0.70 ± 0.73	0.71
	Firmicutes	23.42 ± 14.64	29.90 ± 21.27	0.44
	Fusobacteria	6.50 ± 6.16	6.28 ± 5.88	0.90
	Patescibacteria	0.16 ± 0.34	0.12 ± 0.17	0.43
	Proteobacteria	43.41 ± 37.59	38.01 ± 32.76	0.96
	Spirochaetes	0.07 ± 0.13	0.22 ± 0.66	0.32
	Tenericutes	0.20 ± 0.41	0.09 ± 0.15	0.89
**Class**	Actinobacteria	9.41 ± 18.76	6.08 ± 3.01	0.27
	Alphaproteobacteria	1.83 ± 5.49	0.03 ± 0.08	0.35
	Bacilli	19.19 ± 12.64	27.55 ± 19.85	0.27
	Bacteroidia	15.42 ± 16.51	18.25 ± 12.66	0.83
	Campylobacteria	1.04 ± 1.21	0.70 ± 0.73	0.71
	Clostridia	3.95 ± 4.47	2.17 ± 2.63	0.65
	Coriobacteriia	0.36 ± 0.81	0.33 ± 0.40	0.30
	Fusobacteriia	6.50 ± 6.16	6.28 ± 5.88	0.90
	Gammaproteobacteria	41.57 ± 36.08	37.96 ± 32.75	0.96
	Gracilibacteria	0.15 ± 0.34	0.11 ± 0.16	0.42
	Mollicutes	0.20 ± 0.41	0.09 ± 0.15	0.89
	Negativicutes	0.28 ± 0.60	0.19 ± 0.21	0.71
	Spirochaetia	0.07 ± 0.13	0.22 ± 0.66	0.32
**Order**	Absconditabacteriales (SR1)	0.15 ± 0.34	0.11 ± 0.16	0.42
	Actinomycetales	1.14 ± 1.67	2.05 ± 2.02	0.23
	Bacillales	4.49 ± 6.89	1.99 ± 1.96	0.90
	Bacteroidales	15.06 ± 16.26	15.62 ± 11.84	0.90
	Betaproteobacteriales	34.48 ± 35.85	33.26 ± 33.83	0.84
	Campylobacterales	1.04 ± 1.21	0.70 ± 0.73	0.71
	Caulobacterales	1.63 ± 4.89	0.01 ± 0.02	0.35
	Clostridiales	3.95 ± 4.47	2.17 ± 2.63	0.65
	Coriobacteriales	0.36 ± 0.81	0.33 ± 0.40	0.30
	Corynebacteriales	0.05 ± 0.07	0.30 ± 0.43	0.17
	Flavobacteriales	0.30 ± 0.31	2.62 ± 3.20	***0.03**
	Fusobacteriales	6.50 ± 6.16	6.28 ± 5.88	0.90

	Lactobacillales	14.70 ± 10.16	25.56 ± 18.42	0.18
	Micrococcales	8.21 ± 18.56	3.72 ± 2.31	0.35
	Mycoplasmatales	0.16 ± 0.29	0.09 ± 0.15	0.89
	Pasteurellales	2.53 ± 2.65	4.59 ± 4.43	0.27
	Pseudomonadales	4.55 ± 13.59	0.04 ± 0.08	0.49
	Rhizobiales	0.20 ± 0.59	0 ± 0	0.34
	Selenomonadales	0.28 ± 0.60	0.19 ± 0.21	0.71
	Spirochaetales	0.07 ± 0.13	0.22 ± 0.66	0.32
**Family**	Actinomycetaceae	1.14 ± 1.67	2.05 ± 2.02	0.23
	Aerococcaceae	0.02 ± 0.04	0.61 ± 1.35	0.05
	Atopobiaceae	0.36 ± 0.81	0.31 ± 0.38	0.30
	Bacteroidaceae	0.15 ± 0.43	0 ± 0	0.34
	Burkholderiaceae	0.10 ± 0.16	0.85 ± 1.94	0.08
	Campylobacteraceae	1.03 ± 1.21	0.70 ± 0.73	0.71
	Caulobacteraceae	1.63 ± 4.89	0.01 ± 0.02	0.35
	Corynebacteriaceae	0.05 ± 0.07	0.29 ± 0.42	0.17
	Family XI	1.36 ± 1.30	2.18 ± 2.09	0.30
	Family XIII	0.67 ± 1.06	0.21 ± 0.35	0.77
	Flavobacteriaceae	0.27 ± 0.29	2.47 ± 3.02	***0.04**
	Fusobacteriaceae	4.67 ± 3.78	3.60 ± 3.94	0.77
	Lachnospiraceae	1.32 ± 1.53	0.99 ± 1.25	0.90
	Lactobacillaceae	0.37 ± 1.07	0.02 ± 0.05	0.64
	Leptotrichiaceae	1.83 ± 3.42	2.68 ± 2.71	0.23
	Micrococcaceae	8.15 ± 18.40	3.72 ± 2.31	0.35
	Moraxellaceae	4.53 ± 13.59	0.03 ± 0.08	0.86
	Multi-affiliation	1.17 ± 0.98	2.59 ± 1.79	***0.04**
	Mycoplasmataceae	0.16 ± 0.29	0.09 ± 0.15	0.89
	Neisseriaceae	34.39 ± 35.87	32.41 ± 34.11	0.96
	Pasteurellaceae	2.53 ± 2.65	4.59 ± 4.43	0.27
	Peptostreptococcaceae	0.96 ± 1.47	0.59 ± 0.92	0.77
	Porphyromonadaceae	3.99 ± 6.58	6.04 ± 5.85	0.38
	Prevotellaceae	10.13 ± 12.29	8.94 ± 9.19	0.96
	Rhizobiaceae	0.20 ± 0.59	0 ± 0	0.34
	Rikenellaceae	0.23 ± 0.58	0.04 ± 0.08	0.96
	Ruminococcaceae	0.66 ± 0.97	0.16 ± 0.20	0.30
	Spirochaetaceae	0.07 ± 0.13	0.22 ± 0.66	0.32
	Staphylococcaceae	3.35 ± 7.28	0.009 ± 0.02	0.57
	Streptococcaceae	13.14 ± 9.83	22.34 ± 17.13	0.24
	Tannerellaceae	0.22 ± 0.26	0.50 ± 1.05	0.96
	Unknown	0.24 ± 0.34	0.16 ± 0.16	0.83
	Veillonellaceae	0.28 ± 0.60	0.19 ± 0.21	0.71
**Genus**	*Abiotrophia*	0.02 ± 0.04	0.61 ± 1.35	0.05
	Acinetobacter	4.53 ± 13.59	0 ± 0	0.14
	Actinobacillus	0.04 ± 0.13	0.37 ± 0.69	0.18
	Actinomyces	1.14 ± 1.67	2.05 ± 2.02	0.23

	Aggregatibacter	0.14 ± 0.25	0.78 ± 1.56	0.23
	Alloprevotella	1.49 ± 2.43	0.66 ± 0.82	0.77
	Allorhizobium-Neorhizobium-Pararhizobium-Rhizobium	0.20 ± 0.59	0 ± 0	0.34
	Atopobium	0.36 ± 0.82	0.31 ± 0.38	0.30
	Bacteroides	0.15 ± 0.44	0 ± 0	0.34
	Bergeyella	0.03 ± 0.03	0.15 ± 0.24	0.18
	Brevundimonas	1.63 ± 4.89	0 ± 0	0.34
	Campylobacter	1.04 ± 1.21	0.70 ± 0.73	0.71
	Capnocytophaga	0.27 ± 0.29	2.47 ± 3.02	***0.04**
	Catonella	0.22 ± 0.26	0.09 ± 0.10	0.56
	Corynebacterium	0.05 ± 0.07	0.29 ± 0.43	0.17
	Dialister	0.28 ±0.59	0.18 ± 0.20	0.80
	F0058	0.11 ± 0.16	0.06 ± 0.09	0.70
	Filifactor	0.82 ± 1.40	0.51 ± 0.94	0.90
	Fusobacterium	4.67 ± 3.79	3.60 ± 3.94	0.77
	Gemella	1.14 ± 1.20	1.98 ± 1.97	0.20
	Haemophilus	2.35 ± 2.43	3.45 ± 3,02	0.34
	Kingella	0.06 ± 0.09	0,43 ± 0.99	0.24
	Lachnoanaerobaculum	0.27 ± 0.50	0.17 ± 0.17	0.86
	Lactobacillus	0.37 ± 1.07	0.02 ± 0.05	0.64
	Lautropia	0.08 ± 0.14	0.78 ± 1.85	0.21
	Leptotrichia	1.77 ± 3.44	2.66 ± 2.72	0.26
	Multi-affiliation	1.17 ± 0.98	2.59 ± 1.79	***0.04**
	Mycoplasma	0.16 ± 0.29	0.09 ± 0.15	0.89
	Neisseria	34.31 ± 35.90	31.90 ± 34.47	0.96
	Oribacterium	0.28 ± 0.38	0.31 ± 0.30	0.74
	Parvimonas	0.22 ± 0.26	0.20 ± 0.30	0.90
	Peptostreptococcus	0.12 ± 0.13	0.02 ±0.02	0.10
	Porphyromonas	4.00 ± 6.58	6.04 ± 5.85	0.38
	Prevotella	1.61 ± 2.02	1.46 ± 1.35	0.83
	Prevotella 2	0.08 ± 0.12	0.15 ± 0.17	0.30
	Prevotella 6	0.47 ± 0.81	0.42 ± 0.53	0.96
	"Prevotella 7"	6.23 ± 8.71	6.25 ± 7.20	0.96
	Rikenellaceae RC9 gut group	0.23 ± 0.58	0.04 ± 0.08	0.96
	Rothia	8.15 ± 18.40	3.69 ± 2.32	0.35
	Ruminococcaceae UCG-014	0.66 ± 0.97	0.16 ± 0.20	0.30
	Staphylococcus	3.35 ± 7.28	0.009 ± 0.02	0.57
	Stomatobaculum	0.43 ± 0.58	0.33 ± 0.70	0.93
	Streptococcus	13.09 ± 9.81	22.34 ± 17.13	0.24
	Tannerella	0.22 ± 0.26	0.50 ± 1.05	0.96
	Treponema 2	0.07 ± 0.13	0.22 ± 0.66	0.32
	Unknown	0.63 ± 0.72	0.25 ± 0.20	0.66
	[Eubacterium] brachy group	0.14 ± 0.30	0.05 ± 0.09	0.74
	[Eubacterium] nodatum group	0.28 ± 0.53	0.08 ± 0.13	0.64

<u>Table S2:</u> Comparison between the Relative abundance (%) in oral microbiota of Obese males (OM) BMI>30 (n=5) and Obese females (OF) BMI>30 (n=5). Data as mean ± SD, *p<0.05, **p< 0.001, ***p< 0.0001 unpaired Mann-Whitney test. Significant p-values have been highlighted in bold.

Relative abundance (%) in oral microbiota	Microbiota parameters	Obese males (OM) BMI>30 (n=5)	Obese females (OF) BMI>30 (n=5)	p-value
	α-diversity by the Shannon index	1.36 ± 0.89	1.26 ± 0.27	0.09
	α-diversity by the Chao 1 index	26.41 ± 11.21	39.45 ± 3.74	***0.03**
**Phylum**	Actinobacteria	5.59 ± 2.71	7.23 ± 2.99	0.31
	Bacteroidetes	12.35 ± 12.71	24.16 ± 10.57	0.09
	Epsilonbacteraeota	0.32 ± 0.47	1.08 ± 0.80	0.09
	Firmicutes	13.82 ± 11.71	45.98 ± 15.32	****0.007**
	Fusobacteria	4.42 ± 6.43	8.14 ± 5.27	0.29
	Patescibacteria	0.18 ± 0.23	0.07 ± 0.05	0.99
	Proteobacteria	63.31 ± 25.87	12.72 ± 12.05	****0.007**
	Spirochaetes	0 ± 0	0.45 ± 0.93	***0.02**
	Tenericutes	0.01 ± 0.02	0.18 ± 0.19	0.07
**Class**	Actinobacteria	5.44 ± 2.73	6.72 ± 3.46	0.42
	Bacilli	12.99 ± 11.35	42.11 ± 15.10	***0.03**
	Bacteroidia	12.35 ± 12.71	24.16 ± 10.57	0.09
	Campylobacteria	0.32 ± 0.47	1.08 ± 0.80	0.09
	Clostridia	0.82 ± 0.95	3.52 ± 3.18	0.09
	Coriobacteriia	0.15 ± 0,20	0.51 ± 0.48	0.29
	Fusobacteriia	4.42 ± 6.43	8.14 ± 5.27	0.29
	Gammaproteobacteria	63.28 ± 25.82	12.66 ± 12.09	****0.008**
	Gracilibacteria	0.17 ± 0.22	0.06 ± 0.05	0.83
	Mollicutes	0.01 ± 0.02	0.18 ± 0.19	0.06
	Negativicutes	0.02 ± 0.02	0.36 ± 0.16	***0.01**
	Spirochaetia	0 ± 0	0.45 ± 0.93	***0.02**
**Order**	Absconditabacteriales (SR1)	0.17 ±0.22	0.06 ± 0.05	0.83
	Actinomycetales	0.73 ± 0.62	3.38 ± 2.10	***0.05**
	Bacillales	0.92 ± 0.89	3.05 ± 2.24	0.15
	Bacteroidales	11.17 ± 11.31	20.07 ± 11.74	0.14
	Betaproteobacteriales	58.54 ± 30.30	7.99 ± 7.75	****0.008**
	Campylobacterales	0.32 ± 0.47	1.08 ± 0.80	0.09
	Cardiobacteriales	0.005 ± 0.007	0.11 ± 0.14	***0.01**
	Clostridiales	0.82 ± 0.95	3.52 ± 3.18	0.09
	Coriobacteriales	0.15 ± 0.20	0.51 ± 0.48	0.20
	Corynebacteriales	0.02 ± 0.03	0.58 ± 0.46	***0.01**
	Flavobacteriales	1.18 ± 1.57	4.07 ± 3.92	0.20
	Fusobacteriales	4.42 ± 6.43	8.14 ± 5.27	0.29
	Lactobacillales	12.07 ± 10.48	39.05 ± 14.09	***0.01**

	Micrococcales	4.69 ± 2.57	2.74 ± 1.74	0.31
	Mycoplasmatales	0.01 ± 0.02	0.18 ± 0.19	0.06
	Pasteurellales	4.64 ± 5.08	4.55 ± 4.29	0.99
	Selenomonadales	0.02 ± 0.02	0.36 ± 0.16	***0.01**
	Spirochaetales	0 ± 0	0.45 ± 0.93	***0.02**
**Family**	Actinomycetaceae	0.73 ± 0.62	3.38 ± 2.10	***0.05**
	Aerococcaceae	0.06 ± 0.06	1.15 ± 1.83	0.75
	Atopobiaceae	0.15 ± 0.20	0.47 ± 0.46	0.29
	Burkholderiaceae	0.34 ± 0.44	1.35 ± 2.77	0.99
	Campylobacteraceae	0.32 ± 0.47	1.08 ± 0.80	0.09
	Cardiobacteriaceae	0.01 ± 0.01	0.11 ± 0.14	***0.01**
	Corynebacteriaceae	0.02 ± 0.03	0.57 ± 0.47	***0.01**
	Family XI	0.91 ± 0.91	3.44 ± 2.24	***0.03**
	Family XIII	0.02 ± 0.03	0.39 ± 0.43	***0.01**
	Flavobacteriaceae	1.08 ±1.50	3.86 ± 3.66	0.21
	Fusobacteriaceae	2.30 ± 3.95	4.89 ± 3.89	0.21
	Lachnospiraceae	0.53 ± 0.61	1.45 ± 1.62	0.29
	Leptotrichiaceae	2.12 ± 2.74	3.25 ± 2.85	0.40
	Micrococcaceae	4.69 ± 2.57	2.74 ± 1.74	0.31
	Multi-affiliation	1.44 ± 1.00	3.74 ± 1.70	***0.03**
	Mycoplasmataceae	0.01 ± 0.02	0.18 ± 0.19	0.06
	Neisseriaceae	58.20 ± 30.47	6.63 ± 5.27	****0.008**
	Paludibacteraceae	0.007 ± 0.02	0.11 ± 0.11	***0.01**
	Pasteurellaceae	464 ± 5.08	4.55 ± 4.29	0.99
	Peptostreptococcaceae	0.15 ± 0.24	1.02 ± 1.17	0.09
	Porphyromonadaceae	6.16 ± 7.61	5.91 ± 4.35	0.83
	Prevotellaceae	4.95 ± 7.28	12.93 ± 9.87	0.14
	Rikenellaceae	0.001 ± 0.001	0.08 ± 0.11	****0.009**
	Ruminococcaceae	0.09 ± 0.19	0.22 ± 0.21	0.50
	Spirochaetaceae	0 ± 0	0.45 ± 0.93	***0.02**
	Streptococcaceae	10.55 ± 10.42	34.12 ± 14.29	***0.05**
	Tannerellaceae	0.04 ± 0.09	0.96 ± 1.40	0.06
	Unknown	0.19 ± 0.21	0.14 ± 0.12	0.99
	Veillonellaceae	0.02 ± 0.02	0.36 ± 0.16	***0.01**
	Weeksellaceae	0.10 ± 0.09	0.21 ± 0.34	0.99
**Genus**	*Abiotrophia*	0.06 ± 0.06	1.15 ± 1.83	0.75
	Actinobacillus	0.72 ± 0.97	0.01 ± 0.02	0.15
	Actinomyces	0.73 ± 0.62	3.38 ± 2.10	***0.05**
	Aggregatibacter	0.27 ± 0.54	1.29 ± 2.13	0.29
	Alloprevotella	0.84 ± 1.13	0.48 ± 0.41	0.67
	Atopobium	0.15 ± 0.20	0.47 ± 0.46	0.29
	Bergeyella	0.10 ± 0.09	0.21 ± 0.34	0.99
	Campylobacter	0.33 ± 0.47	1.08 ± 0.80	0.09
	Capnocytophaga	1.08 ± 1.50	3.86 ± 3.66	0.20
	Cardiobacterium	0.005 ± 0.007	0.11 ± 0.14	***0.01**

	Catonella	0.05 ± 0.09	0.13 ± 0.10	0.29
	Corynebacterium	0.02 ± 0.03	0.57 ± 0.47	***0.01**
	Dialister	0.02 ± 0.02	0.33 ± 0.15	***0.01**
	Eikenella	0.07 ± 0.09	0.11 ± 0.13	0.52
	F0058	0.007 ± 0.01	0.11 ± 0.11	***0.01**
	Filifactor	0.03 ± 0.03	0.99 ± 1.18	***0.01**
	Fusobacterium	2.30 ± 3.95	4.89 ± 3.89	0.20
	Gemella	0.91 ± 0.91	3.05 ± 2.24	0.15
	Haemophilus	3.64 ± 3.89	3.25 ± 2.31	0.99
	Kingella	0.08 ± 0.10	0.77 ± 1.37	0.20
	Lachnoanaerobaculum	0.16 ± 0.21	0.18 ± 0.14	0.83
	Lautropia	0.28 ± 0.47	1.27 ± 2.62	0.83
	Leptotrichia	2.08 ± 2.77	3.25 ± 2.85	0.40
	Moryella	0 ± 0	0.14 ± 0.24	***0.02**
	Multi-affiliation	1.44 ± 1.00	3.74 ± 1.70	***0.03**
	Mycoplasma	0.01 ± 0.02	0.18 ± 0.19	0.06
	Neisseria	58.05 ± 30.64	5.75 ± 5.03	****0.008**
	Oribacterium	0.22 ± 0.25	0.41 ± 0.34	0.67
	Parvimonas	0.006 ± 0.005	0.40 ± 0.33	***0.01**
	Porphyromonas	6.16 ± 7.61	5.91 ± 4.35	0.83
	Prevotella	0.86 ± 1.10	2.07 ± 1.40	0.14
	Prevotella 2	0.10 ± 0.19	0.19 ± 0.16	0.29
	Prevotella 6	0.13 ± 0.28	0.70 ± 0.59	0.10
	Prevotella 7	3.02 ± 5.70	9.47 ± 7.62	0.14
	Rikenellaceae RC9 gut group	0.001 ± 0.002	0.08 ± 0.11	***0.009**
	Rothia	4.65 ± 2.61	2.74 ± 1.74	0.31
	Ruminococcaceae UCG-014	0.09 ± 0.19	0.22 ± 0.21	0.50
	Stomatobaculum	0.09 ± 0.11	0.56 ± 0.98	0.39
	Streptococcus	10.55 ± 10.42	34.12 ± 14.29	***0.05**
	Tannerella	0.04 ± 0.09	0.96 ± 1.40	0.06
	Treponema 2	0 ± 0	0.45 ± 0.93	***0.02**
	Unknown	0.24 ± 0.21	0.26 ± 0.22	0.99
	Veillonella	0.001 ± 0.002	0.02 ± 0.02	****0.009**

<u>Table S3:</u> Comparison between the Relative abundance (%) in oral microbiota of Normo-weighted females (NWF) 20<BMI<25 (n=4) and Obese females (OF) BMI>30 (n=5). Data as mean ± SD, *p<0.05, **p< 0.001, ***p< 0.0001 unpaired Mann-Whitney test. Significant p-values have been highlighted in bold.

Relative abundance (%) in oral microbiota	Microbiota parameters	Normo-weighted females (NWF) 20<BMI<25 (n=4)	Obese females (OF) BMI>30 (n=5)	p-value
	α-diversity by the Shannon index	1.47 ± 0.96	2.26 ± 0.27	0.11
	α-diversity by the Chao 1 index	31.69 ± 15.28	39.45 ± 3.74	0.55
**Phylum**	Actinobacteria	2.06 ± 2.03	7.23 ± 2.99	***0.03**
	Bacteroidetes	11.75 ± 16.89	24.16 ± 10.57	0.19
	Epsilonbacteraeota	0.54 ± 0.42	1.08 ± 0.80	0.55
	Firmicutes	21.67 ± 20.34	45.98 ± 15.32	0.11
	Fusobacteria	4.57 ± 3.17	8.14 ± 5.27	0.55
	Proteobacteria	58.83 ± 41.57	12.72 ± 12.05	0.28
	Spirochaetes	0.12 ± 0.20	0.45 ± 0.93	0.80
	Tenericutes	0.37 ± 0.61	0.18 ± 0.19	0.90
**Class**	Actinobacteria	1.95 ± 1.87	6.72 ± 3.46	0.11
	Alphaproteobacteria	4.12 ± 8.23	0.06 ± 0.11	0.53
	Bacilli	17.65 ± 15.45	42.11 ± 15.10	0.06
	Bacteroidia	11.75 ± 16.89	24.16 ± 10.57	0.19
	Campylobacteria	0.54 ± 0.43	1.08 ± 0.80	0.55
	Clostridia	3.88 ± 5.24	3.52 ± 3.18	0.90
	Coriobacteriia	0.11 ± 0.19	0.51 ± 0.48	0.11
	Fusobacteriia	4.57 ± 3.17	8.14 ± 5.27	0.55
	Gammaproteobacteria	54.72 ± 39.86	12.66 ± 12.09	0.28
	Mollicutes	0.37 ± 0.61	0.18 ± 0.19	0.90
	Negativicutes	0.14 ± 0.19	0.36 ± 0.16	0.19
	Spirochaetia	0.12 ± 0.20	0.45 ± 0.93	0.80
**Order**	Actinomycetales	0.49 ± 0.62	3.38 ± 2.10	***0.03**
	Bacillales	1.03 ± 0.93	3.05 ± 2.24	0.11
	Bacteroidales	11.46 ± 16.81	20.07 ± 11.74	0.55
	Betaproteobacteriales	43.60 ± 42.64	7.99 ± 7.75	0.28
	Campylobacterales	0.54 ± 0.43	1.08 ± 0.80	0.55
	Cardiobacteriales	0.004 ± 0.003	0.11 ± 0.14	***0.01**
	Caulobacterales	3.67 ±7.34	0.02 ± 0.03	0.89
	Clostridiales	3.88 ± 5.24	3.52 ± 3.18	0.90
	Coriobacteriales	0.11 ± 0.19	0.51 ± 0.48	0.11
	Corynebacteriales	0.04 ± 0.06	0.58 ± 0.46	***0.03**
	Flavobacteriales	0.24 ± 0.17	4.07 ± 3.92	***0.01**
	Fusobacteriales	4.57 ± 3.17	8.14 ± 5.27	0.55
	Lactobacillales	16.62 ± 14.59	39.05 ± 14.09	0.06
	Micrococcales	1.40 ± 1.20	2.74 ± 1.74	0.28

	Mycoplasmatales	0.27 ± 0.42	0.18 ± 0.19	0.90
	Pasteurellales	0.91 ± 1.37	4.55 ± 4.29	0.06
	Pseudomonadales	10.21 ± 20.39	0.01 ± 0.008	0.90
	Rhizobiales	0.44 ± 0.89	0 ± 0	0.37
	Selenomonadales	0.14 ± 0.19	0.36 ± 0.16	0.19
	Spirochaetales	0.12 ± 0.20	0.45 ± 0.93	0.80
**Family**	Acholeplasmataceae	0.10 ± 0.19	0 ± 0	0.37
	Actinomycetaceae	0.49 ± 0.62	3.38 ± 2.10	***0.03**
	Aerococcaceae	0.02 ± 0.04	1.15 ± 1.83	0.20
	Atopobiaceae	0.11 ± 0.19	0.48 ± 0.46	0.11
	Burkholderiaceae	0.03 ± 0.06	1.35 ± 2.77	0.11
	Campylobacteraceae	0.54 ± 0.43	1.08 ± 0.80	0.55
	Cardiobacteriaceae	0.004 ± 0.003	0.11 ± 0.14	***0.01**
	Caulobacteraceae	3.67 ± 7.34	0.02 ± 0.03	0.89
	Clostridiales vadinBB60 group	0.17 ± 0.33	0.01 ± 0.02	0.89
	Corynebacteriaceae	0.04 ± 0.06	0.57 ± 0.47	***0.03**
	Family XI	1.39 ± 1.13	3.44 ± 2.24	0.19
	Family XIII	0.76 ± 1.31	0.39 ± 0.43	0.73
	Flavobacteriaceae	0.22 ± 0.15	3.86 ± 3.66	***0.01**
	Fusobacteriaceae	4.19 ± 2.92	4.89 ± 3.89	0.99
	Lachnospiraceae	0.99 ± 1.17	1.45 ±1.62	0.41
	Leptotrichiaceae	0.38 ± 0.42	3.25 ± 2.85	***0.01**
	Micrococcaceae	1.39 ± 1.20	2.74 ± 1.74	0.28
	Moraxellaceae	10.20 ± 20.39	0.0006 ± 0.001	0.86
	Multi-affiliation	0.73 ± 0.52	3.74 ± 1.70	***0.01**
	Mycoplasmataceae	0.27 ± 0.42	0.18 ± 0.19	0.91
	Neisseriaceae	43.57 ± 42.67	6.63 ± 5.27	0.19
	Paludibacteraceae	0.19 ± 0.23	0.11 ± 0.11	0.90
	Pasteurellaceae	0.91 ± 1.37	4.55 ± 4.30	0.06
	Peptostreptococcaceae	1.37 ± 1.99	1.02 ± 1.17	0.99
	Porphyromonadaceae	5.13 ± 9.33	5.91 ± 4.35	0.28
	Prevotellaceae	5.09 ± 5.81	12.93 ± 9.87	0.28
	Rhizobiaceae	0.44 ± 0.89	0 ± 0	0.37
	Rikenellaceae	0.46 ± 0.88	0.08 ± 0.11	0.90
	Ruminococcaceae	0.18 ± 0.31	0.22 ± 0.21	0.90
	Spirochaetaceae	0.12 ± 0.20	0.45 ± 0.93	0.80
	Streptococcaceae	15.86 ± 14.21	34.12 ± 14.29	0.11
	Tannerellaceae	0.18 ± 0.20	0.96 ± 1.40	0.35
	Unknown	0.19 ± 0.21	0.14 ± 0.12	0.99
	Veillonellaceae	0.14 ± 0.19	0.36 ± 0.16	0.19
	Weeksellaceae	0.02 ± 0.01	0.21 ± 0.37	0.14
**Genus**	*Abiotrophia*	0.02 ± 0.04	1.15 ± 1.83	0.20
	Acinetobacter	10.20 ± 20.39	0 ± 0	0.37
	Actinomyces	0.49 ± 0.62	3.38 ± 2.10	***0.03**
	Aggregatibacter	0.03 ± 0.05	1.29 ± 2.13	0.10

	Alloprevotella	1.17 ± 1.84	0.48 ± 0.41	0.90
	Atopobium	0.11 ± 0.19	0.47 ± 0.46	0.11
	Bergeyella	0.02 ± 0.01	0.21 ± 0.34	0.14
	Brevundimonas	3.67 ± 7.34	0 ± 0	0.37
	Campylobacter	0.54 ± 0.43	1.08 ± 0.80	0.55
	Capnocytophaga	0.22 ± 0.15	3.86 ± 3.66	***0.01**
	Cardiobacterium	0.004 ± 0.003	0.11 ± 0.14	***0.01**
	Catonella	0.31 ± 0.27	0.13 ± 0.10	0.32
	Corynebacterium	0.04 ± 0.06	0.57 ± 0.47	***0.03**
	Dialister	0.14 ± 0.19	0.33 ± 0.15	0.19
	Eikenella	0.03 ± 0.04	0.11 ± 0.13	0.38
	F0058	0.19 ± 0.23	0.11 ± 0.11	0.90
	Filifactor	1.21 ± 1.94	0.99 ± 1.18	0.73
	Fusobacterium	4.19 ± 2.92	4.89 ± 3.89	0.99
	Gemella	1.03 ± 0.93	3.05 ± 2.24	0.11
	Haemophilus	0.87 ± 1.31	3.25 ± 2.31	0.11
	Kingella	0.05 ± 0.04	0.77 ± 1.37	0.21
	Lachnoanaerobaculum	0.18 ± 0.29	0.19 ± 0.14	0.80
	Lautropia	0.03 ± 0.06	1.27 ± 2.62	0.20
	Leptotrichia	0.26 ± 0.35	3.25 ± 2.85	***0.01**
	Moryella	0.02 ± 0.03	0.14 ± 0.24	0.53
	Multi-affiliation	0.73 ± 0.52	3.74 ± 1.70	***0.01**
	Mycoplasma	0.27 ± 0.42	0.18 ± 0.19	0.90
	Neisseria	43.50 ± 42.70	5.75 ± 5.03	0.19
	Oribacterium	0.17 ± 0.30	0.41 ± 0.34	0.46
	Parvimonas	0.37 ± 0.31	0.40 ± 0.33	0.90
	Peptostreptococcus	0.16 ± 0.14	0.02 ± 0.02	0.21
	Porphyromonas	5.13 ± 9.33	5.91 ± 4.35	0.28
	Prevotella	0.61 ± 0.53	2.07 ± 1.40	0.11
	Prevotella 2	0.07 ± 0.10	0.19 ± 0.16	0.19
	Prevotella 6	0.06 ± 0.09	0.70 ± 0.59	0.14
	Prevotella 7	2.63 ± 4.30	9.47 ± 7.62	0.19
	Rikenellaceae RC9 gut group	0.46 ± 0.88	0.08 ± 0.11	0.90
	Rothia	1.39 ± 1.20	2.74 ± 1.74	0.28
	Ruminococcaceae UCG-014	0.18 ± 0.31	0.22 ± 0.21	0.90
	Stomatobaculum	0.29 ± 0.54	0.56 ± 0.98	0.46
	Streptococcus	15.86 ± 14.21	34.12 ± 14.29	0.11
	Tannerella	0.18 ± 0.20	0.96 ± 1.40	0.32
	Treponema 2	0.12 ± 0.20	0.45 ± 0.93	0.80
	Unknown	0.82 ± 1.00	0.26 ± 0.22	0.99
	[Eubacterium] brachy group	0.23 ± 0.45	0.10 ± 0.11	0.62
	[Eubacterium] nodatum group	0.10 ± 0.19	0.15 ± 0.16	0.53
	[Eubacterium] saphenum group	0.25 ± 0.37	0.08 ± 0.16	0.68
